# Factors influencing Chinese EFL students’ online learning anxiety in the post-COVID-19 era

**DOI:** 10.1016/j.heliyon.2024.e26112

**Published:** 2024-02-14

**Authors:** Renzhong Peng, Shiying Wang, Na Liu

**Affiliations:** a School of Foreign Languages, Huazhong University of Science and Technology, Wuhan, Hubei, China; b School of Foreign Languages, Jingchu University of Technology, Jingmen, Hubei, China

**Keywords:** Online learning anxiety, Chinese EFL students, College English course, Post-COVID-19 era

## Abstract

Online learning has evolved as an attractive and viable option for education, yet there is a need for further research to investigate the factors contributing to students' online learning anxiety regarding the college English course. Based on Keegan's distance education framework, this study examines the factors impacting online learning anxiety among English as foreign language (EFL) students in the Chinese context during the post-COVID-19 era. Data were collected from 899 EFL students across different regions of China through an online survey. Follow-up interviews with ten students provided additional insights into the association between online English learning and anxiety. The collected data underwent descriptive analysis, exploratory factor analysis, reliability analysis, and multiple linear regression analysis to examine the relationship between online learning anxiety and the identified factors. The results of our study indicate that many Chinese EFL students experienced different degrees of anxiety, ranging from mild to moderate or severe. Moreover, online learning anxiety among Chinese EFL students was positively predicted by a lack of learning motivation, separation from instructors, separation from peers, and technological challenges, while a lack of two-way communication negatively predicted it. The findings underscore the importance of taking effective measures and offering psychological guidance for Chinese EFL students to alleviate anxiety and facilitate their successful adaptation to the new normal of online learning.

## 1 Introduction

Given the technological advancements and the transformative impact of the COVID-19 pandemic, online learning has become an appealing method of learning and will become a viable option in the foreseeable future [1,2]. However, using digital devices as intermediaries for communication in online learning diminishes the meaningfulness of non-verbal communication between individuals [3]. As a result, online learning anxiety arises because of the absence of interpersonal interaction under the circumstances [4]. Furthermore, the sudden transition to urgently developed online courses due to the COVID-19 pandemic has imposed considerable pressure on students [5], substantially exacerbating their online learning anxiety.

Previous studies have examined the impact of students' online learning anxiety and the challenges associated with online learning [6]. Additionally, a focus has been placed on exploring language learning anxiety in online contexts [7]. These studies have delved into various aspects of language learning anxiety, including writing and speaking anxiety in an online learning environment [8], as well as the effects of online learning on EFL learners' anxiety [9]. Furthermore, scholars in China have displayed much concern regarding English learning anxiety among college students, exploring multiple dimensions such as speech anxiety [10], writing anxiety [11], test anxiety [12], and fear of negative evaluation in traditional classrooms [13]. Nevertheless, there is a paucity of studies examining the specific factors that may influence EFL students’ learning anxiety within an online learning environment, particularly in China. Therefore, the main objective of this study is to assess online learning anxiety and pinpoint its influencing factors within a cohort of 899 Chinese EFL students in the post-COVID-19 era, employing multilinear regression analysis. This study endeavors to provide valuable academic insights into the impact of these factors on online learning anxiety within the context of the college English course during the post-COVID-19 period. The findings of this study hold crucial implications for both theoretical understanding and practical application in addressing and mitigating online learning anxiety among EFL students in the college English course.

## 2 Literature review

### 2.1 Online learning

In the 1990s, the educational landscape underwent significant changes due to the ever-expanding influence of technology [14]. One of the most notable developments during this period was the extensive implementation of online learning across diverse contexts. The term “online learning” was initially introduced in 1995 and has since become synonymous with various terms such as e-learning, online education, distance education, and online courses [15]. Despite the ambiguity of the terminology, researchers in this field are seeking some common ground. Online learning can be conceptualized as integrating two major domains: learning and technology. Learning pertains to the cognitive procedure of acquiring knowledge, while technology facilitates this process [16].

The literature on online learning and distance education has rapidly expanded in the past few years. Researchers have made numerous attempts to define distance education [17], with prominent theories proposed by Keegan [18], Peters (1983), Holmberg (1986), and Moore [19] playing a crucial role in defining distance education as a subdiscipline of education [20]. These theories remain relevant today and are essential for scholars and practitioners seeking to comprehend the concept of “distance education” in its various forms [20]. For instance, Keegan was among the pioneering scholars who systematically compared online education and traditional education to distinguish the unique characteristics of online education [18].

Amid the global COVID-19 pandemic, online learning has received significant attention from the academic community as the education sector worldwide has shifted to virtual learning. The shift to online learning presents numerous advantages for students, including the flexibility to learn at a self-determined pace and maintain continuous access to learning opportunities [21]. Therefore, it is successfully used in both academia and industry with positive outcomes. However, compared to face-to-face learning, online learning poses many challenges that need to be addressed, including maintaining students' motivation and engagement [22], technical issues [23], physical distance, lack of feedback, and limited interactivity [24]. These challenges can have both immediate and long-term impacts on students’ physiological and psychological well-being, as well as their academic achievements and overall lives [25].

### 2.2 Learning anxiety

Anxiety is a complex and abstract concept studied from various perspectives. Initially, behaviorism and cognitive approaches contributed to our understanding of anxiety. Sigmund Freud directed attention to anxiety as the fundamental question for understanding emotional and psychological disorders [26]. Based on its distinct experiential and physiological qualities, Freud distinguished anxiety from other negative emotional states, such as anger, grief, or sorrow. Furthermore, anxiety has been a crucial research variable for a long time, with a fruitful history [27] dating back to Mowrer's classic study, which argued that anxiety is a learned response to conditioned stimuli premonitory of injury or pain situations [28]. In the early days, psychologists and neurologists focused on trait anxiety. As computers and the internet became widely used, researchers began investigating various types of anxiety in the digital era, including computer anxiety [29], internet anxiety [30], and online learning anxiety [31]. As a result, anxiety has emerged as a prominent subject of research focus across various fields, including psychology, behavioral science, and education.

The existing literature has primarily centered on investigating online learning anxiety across various online settings, such as self-paced online training, online examinations, and synchronous (asynchronous) online courses, particularly within the context of the COVID-19 pandemic. Researchers such as Abdelwahed et al. [7] have employed structural equation modeling analysis to demonstrate that factors such as a lack of time and support, technical problems, and poor technical skills all negatively influence online learning. Moreover, studies have also examined the impact of the pandemic on students’ anxiety [32], the effects of COVID-19 on online learning [33], online motivation, performance, and engagement [34,35], as well as online learning self-efficacy [36].

Foreign language classroom anxiety has been investigated since the 1980s [37]. Previous studies have mainly focused on defining, characterizing, and classifying this type of anxiety [38,39]. Additionally, scholarly investigations have explored the influence of anxiety on the acquisition of foreign languages and the correlation between anxiety and learners' language performance [40]. Numerous studies have indicated that anxiety interferes with learners' cognitive processes, negatively impacting their academic achievement [41] and decreasing performance [42]. The concept of foreign language classroom anxiety was introduced by Horwitz in 1986 [38]. Krasen proposed the “affective filter hypothesis,” suggesting that a mental block known as the “affective filter” can hinder learners' effective utilization of comprehensible input. When learners lack confidence, feel anxious, or become defensive, this filter can be “up.” Higher anxiety levels exacerbate the affective filtering effect, hindering learners’ access to language input and impeding progress in language acquisition [43]. Furthermore, extensive research and measurement have been conducted on foreign language learning anxiety from various perspectives, highlighting its intrinsic connection to the overall effectiveness of foreign language learning [40,44]. While some studies suggest that moderate anxiety contributes to foreign language learning, most research confirms that learning anxiety has detrimental effects. Specifically, anxiety and language learning outcomes have a circular relationship, forming a vicious circle [45].

### 2.3 Students’ anxiety toward the online foreign language course

College students' anxiety and stress are common psychological issues [46]. However, these issues may impact students' quality of life, learning, and academic performance [47]. With the rapid advancement of information technology and the widespread use of online courses, many students experience fear, anxiety, and apprehension about online learning [48] as the multimedia environment differs significantly from traditional classroom settings. Anxiety and learning environments are inextricably linked [41]. Due to the COVID-19 pandemic, many language learning courses are offered online, which may exacerbate students' anxiety and apprehension. Moreover, online teaching and learning will become an inevitable trend in the future. Thus, students’ anxiety related to online learning cannot be ignored.

### 2.4 Theoretical framework

The theoretical underpinning of the current study hinged upon Keegan's distance education framework. Keegan's research identified five elements that comprise a comprehensive definition of distance education, including the separation of teacher and learner through the length of the learning process, the influence of an educational organization, the use of technological media, the provision of two-way communication, and the absence of learning groups [49]. These dimensions helped explore the factors affecting Chinese EFL students' online learning anxiety in the post-COVID-19 era. By adopting this theoretical framework, the researchers effectively explained the factors often identified in online learning contexts.

The preceding review of literature on online learning and foreign language learning anxiety highlights that challenges and factors influencing EFL students' online learning anxiety remain unclear. It is evident that anxiety plays a critical role in the teaching and learning process, and this role needs to be addressed in both theory and practice. While some factors influencing online learning anxiety have been explored [7,50], there is a lack of a systematic framework to investigate the factors and understand the impact on EFL students' online learning anxiety in the context of college English courses in China during the post-COVID-19 pandemic era. Therefore, this study attempts to fill this gap by conducting an in-depth investigation into the anxiety levels of EFL students studying online and examining the factors that affect their online college English learning anxiety. Building upon prior literature and Keegan's distance education framework, the following research questions (RQ) are addressed.

RQ1 What is the overall state of online learning anxiety in the college English course among Chinese EFL students in the post-COVID-19 era?

RQ2 What are the factors affecting Chinese EFL students' online learning anxiety about the college English course in the post-COVID-19 era? RQ3. How do these factors affect Chinese EFL students' online learning anxiety in the college English course in the post-COVID-19 era?

## 3 Research methods

### 3.1 Participants and research context

This study included 899 Chinese EFL students (326 males and 573 females) between the ages of 17 and 21 (*M* = 19.43, *SD* = 1.123). A stratified random sampling method was used to ensure participant representation. This method involved stratifying the sample based on the regions and universities where the EFL students were. The participants were selected from various types of universities, including directly affiliated universities under the Ministry of Education (28.4%), provincial universities (33.7%), local universities (29.9%), and private universities (8.0%) from 15 Chinese provinces or municipalities (see Table 1). This study focused on first and second-year EFL students enrolled in the college English course online during the fall semester of 2022, a period influenced by the pandemic. Juniors and seniors were excluded from the study as college English courses are not offered in China. Follow-up interviews were conducted with ten students to gain further insights into the relationship between online learning and anxiety, as indicated in Table 2.

**Table 1:** Demographic background of participants.

Category	Level	*n*	% (*n* = 899)
Age (years)	17	37	4.10
	18	164	18.20
	19	252	28
	20	265	29.50
	21	181	20.1
Gender	Male	326	36.3
	Female	573	63.7
University	DAUME^a^	255	28.4
	PU^a^	303	33.7
	LU^a^	269	29.9
	pu^a^	72	8.0
Academic year	First year	416	46.3
	Second year	483	53.7
Major	Liberal Arts	327	36.4
	Science and Engineering	346	38.5
	Others	226	25.1
Residence	Urban	239	26.6
	Rural	660	73.4

Note:

a DAUME stands for Directly Affiliated Universities under the Ministry of Education, PU stands for Provincial Universities, LU stands for Local Universities, and pu stands for private universities.

**Table 2:** List of the interviewees.

Participant	Major	Academic Year	Residence
Interviewee-A	Chemistry	First Year	Urban
Interviewee-B	Electronic Information	Second Year	Rural
Interviewee-C	German	Second Year	Urban
Interviewee-D	Japanese	First Year	Urban
Interviewee-E	Chinese Language and Literature	Second Year	Rural
Interviewee-F	Law	First Year	Urban
Interviewee-G	Marketing	First Year	Urban
Interviewee-H	Business Administration	Second Year	Rural
Interviewee-I	Energy	First Year	Rural
Interviewee-J	Law	Second Year	Rural

In this study, approval was obtained from the School of Foreign Languages ethics committee, Huazhong University of Science and Technology (the ethical approval of Research Project Protocol code: #2022-SFL/IRB-0820). Participation was voluntary, and participants could respond at their convenience and according to their schedules. Privacy was ensured, and only those who agreed to participate and share their viewpoints about the online college English course were included.

### 3.2 Measurement instruments

The students completed a survey consisting of three sections: (1) demographic information including age, gender, university, academic year, major, and residence area; (2) the Online Course Learning scale; (3) the measurement tool for anxiety (Generalized Anxiety Disorder-7).

#### 3.2.1 The online course learning scale

The online course learning scale in the present study was adapted from Li, Lan, and Williams' 5-point Likert Scale of Online Course Anxiety [27]. We made some modifications to fit better the unique circumstances of EFL students' online learning experiences in our study. Firstly, the questionnaire was translated into Chinese and adapted accordingly. Secondly, the items were revised by removing phrases beginning with “I am afraid/stressed/nervous” and changing them to more objective statements. For example, “I am afraid that my instructor is so separated from students in my online course that s/he may not know our feelings” was revised to “My instructor is so separated from students in my online course that s/he may not know our feelings.” Thirdly, we added a new dimension (lack of learning motivation) with five items based on previous literature and student interviews. Examples of the newly added items include “I am poorly self-motivated when studying online,” “I don't want to take online English classes,” and “I can't concentrate when studying online.” The revised questionnaire consisted of 24 items, measured on a 5-point Likert scale ranging from 1 (strongly disagree) to 5 (strongly agree). Finally, two experts in course management and second language acquisition reviewed the questionnaire and provided suggestions for improvement in terms of wording and framing. The experts confirmed the content validity of the questionnaire, resulting in a combined rating score of 95% based on standards such as appropriateness, relevance, and clarity. The high rating indicates a reasonably high level of content validity. The internal consistency reliability (Cronbach's α = 0.959) and structural validity (χ^2^/df = 2.941 < 3; RMR = 0.042 < 0.08; GFI = 0.942 > 0.90; AGFI = 0.922 > 0.90; TLI = 0.968 ≥ 0.90; CFI = 0.974 > 0.90; RMSEA = 0.046 < 0.08) of this scale were satisfactory.

#### 3.2.2 Measurement tool for anxiety

The Generalized Anxiety Disorder-7 (GAD-7) scale [51] was used in this study to identify anxiety symptoms among the subjects. It is one of the most reliable measures of generalized anxiety disorder for assessing various populations, including college students [52]. In this study, the instrument consists of seven items, all situated in an online learning environment, describing the core symptoms of anxiety. These items are scored on a 4-point Likert scale, with “0 = not at all”, “1 = several days”, “2 = more than half the days”, and “3 = almost every day”. The total score ranges from 0 to 21, with scores classified as follows: 0–4 indicates no anxiety, 5–9 indicates mild anxiety, 10–14 indicates moderate anxiety, and 15 or higher indicates severe anxiety. The GAD-7 score of 4 was used as the threshold between healthy control and anxiety [53]. The GAD-7 scale demonstrated excellent internal consistency (Cronbach's Alpha = .92) and procedural, criterion, construct, and factorial validity, as established in previous studies [51]. In the present study, the retest Cronbach's Alpha value for the Chinese version of this scale was found to be 0.954, indicating strong internal consistency.

### 3.3 Data collection and data analysis

Data for this study were collected from a questionnaire and semi-structured interviews, launched on January 3, 2023 and continued to 18 January 18, 2023. The questionnaire was distributed to 940 students via the online survey platform “Wenjuanxing.” Out of 940 questionnaires, 899 valid responses were received, resulting in a response rate of 95.6%. The participants completed the questionnaire in approximately 5 min. Data collected through the questionnaire were analyzed using SPSS version 26.

Firstly, exploratory factor analysis was performed on the revised Online Course Learning Scale to extract the main factors and remove any items that did not meet the requirements for factor extraction. Secondly, a reliability analysis was performed to test the internal consistency of the questionnaire. A 24-item questionnaire that had been revised was constructed. Thirdly, descriptive and correlation analyses were performed to examine online learning anxiety and explore the relationship among variables. Furthermore, semi-structured interviews with ten students were conducted to refine the questionnaire design and interpret the questionnaire data results. Each interview lasted approximately 15–20 min and consisted of four open-ended questions.

## 4 Results

### 4.1 Chinese EFL students’ anxiety levels

Table 3 presents the distribution of anxiety levels among Chinese EFL students, including the percentages, means, and standard deviations. The results indicate that 45.9% of the participants reported no anxiety, while 54.1% reported experiencing mild to severe anxiety. Specifically, 35.6% reported mild anxiety, 12.7% reported moderate anxiety, and 5.8% exhibited severe anxiety. The average score for generalized anxiety disorder was 14.202 (*SD* = 5.234).

**Table 3:** Descriptive statistics for the anxiety level of the students (*n* = 899).

Level of anxiety	Minimum	Maximum	Percentage	Mean	Standard Deviation (*SD*)
No anxiety	0	4	45.9	1.110	1.424
Mild anxiety	5	9	35.6	6.930	1.201
Moderate anxiety	10	14	12.7	11.790	1.554
Severe anxiety	15	21	5.8	18.920	2.300
**Total**			100	14.202	5.234

To investigate differences in anxiety levels based on demographic information, the Mann-Whitney test and the Kruskal Wallis Test were conducted. Table 4 presents the univariate analysis of anxiety levels among Chinese EFL students. No evidence supports significant differences in anxiety levels among them based on age, gender, university levels, academic year, or major. However, a significant difference was found in the total anxiety scores of students based on their residence areas (birthplace). Specifically, students from rural areas had higher anxiety levels (median = 6.00, IQR = 7) than those from urban areas (median = 4.00, IQR = 7) (U = 68513.50, N1 = 239, N2 = 660, *p* = .002, two-tailed).

**Table 4:** Demographic characteristics of the respondents’ anxiety levels (*n* = 899).

Category	n (%)					
	No anxiety	Mild anxiety	Moderate anxiety	Severe anxiety	Statistics	*P* value
Age (years)						
17	18 (48.6)	12 (32.4)	5 (13.5)	2 (5.4)	1.548^b^	0.818
18	78 (47.6)	58 (35.4)	19 (11.6)	9 (5.5)		
19	123 (48.8)	83 (32.9)	34 (13.5)	12 (4.8)		
20	115 (43.4)	103 (38.9)	31 (11.7)	16 (6.0)		
21	79 (43.6)	64 (35.4)	25 (13.8)	13 (7.2)		
**Gender**						
Male	162 (49.7)	100 (30.7)	44 (13.5)	20 (6.1)	89315.50^c^	0.270
Female	251 (43.8)	220 (38.4)	70 (12.2)	32 (5.6)		
**University**						
DAUME^a^	111 (43.5)	96 (37.6)	33 (12.9)	15 (5.9)	3.841^b^	0.279
PU^a^	148 (48.8)	104 (34.3)	34 (11.2)	17 (5.6)		
LU^a^	127 (47.2)	93 (34.6)	37 (13.8)	12 (4.5)		
pu^a^	27 (37.5)	27 (37.5)	10 (13.9)	8 (11.1)		
**Academic year**						
First year	203 (48.8)	140 (33.7)	48 (11.5)	25 (6.0)	95551.500^c^	0.201
Second year	210 (43.5)	180 (37.3)	66 (13.7)	27 (5.6)		
**Major**						
Liberal Arts	135 (41.3)	117 (35.8)	51 (15.6)	24 (7.3)	5.175^b^	0.075
Science and Engineering	167 (48.3)	124 (35.8)	40 (11.6)	15 (4.3)		
Others	111 (49.1)	79 (35.0)	23 (10.2)	13 (5.8)		
**Residence**						
Urban	130 (54.4)	69 (28.9)	24 (10.0)	16 (6.7)	68513.500^c^	0.002
Rural	283 (42.9)	251 (38.0)	90 (13.6)	36 (5.5)		

Note:

a DAUME stands for Directly Affiliated Universities under the Ministry of Education, PU stands for Provincial Universities, LU stands for Local Universities, and pu stands for private universities.

b Kruskal Wallis Test.

c Mann-Whitney Test.

### 4.2 Reliability analysis, validity analysis and exploratory factor analysis

Principal component analysis and varimax with Kaiser Normalization were performed to examine the internal structure of the 24-item questionnaire of the students' general situation of online English course learning and conduct factor extraction. The analysis converged after eight times of iteration rotations. The results revealed a high level of internal consistency, with Cronbach's alpha of 0.959. The Kaiser-Meyer-Olkin (KMO) statistic obtained was 0.960 (>0.800), and Bartlett's test of sphericity was significant (χ^2^ = 16948.818, df = 276, *p* = .000 < 0.001), indicating that the sample size and data collection met the requirements for factor analysis. Following the rotation, five latent factors were extracted based on the criteria of an eigenvalue greater than 1.0 and factor loading greater than 0.50. These criteria are considered acceptable for establishing construct validity [54]. In the final version of the scale, students' online course learning state was assessed by five components: lack of learning motivation (5 items, with factor loadings ranging from 0.515 to 0.829, and all factor loadings were significant at *p* < .001; α = 0.879), separation from instructors (4 items, with factor loadings ranging from 0.599 to 0.768, and all factor loadings were significant at *p* < .001; α = 0.858), separation from peers (3 items, with the factor loadings ranging from 0.632 to 0.761, and all factor loadings were significant at *p* < .001; α = 0.852), technological challenges (6 items, with factor loadings ranging from 0.510 to 0.742, and all factor loadings were significant at *p* < .001; α = 0.866), lack of two-way communication (6 items, with factor loadings ranging from 0.579 to 0.819, and all factor loadings were significant at *p* < .001; α = 0.959) (see Table 5, Table 6).

**Table 5:** Reliability and validity analysis results.

Dimension	Cronbach's Alpha	KMO	Items
Lack of Learning Motivation	0.879	0.842	5
Separation from Instructors	0.858	0.792	4
Separation from Peers	0.852	0.727	3
Technological Challenges	0.866	0.842	6
Lack of Two-way Communication	0.949	0.904	6
**Total**	0.959	0.960	24

**Table 6:** EFA results.

Dimension/Item	Factor loading	Eigenvalue	% of Variance
Lack of Learning Motivation (LLM)		4.421	18.421
LLM1	0.818		
LLM2	0.829		
LLM3	0.737		
LLM4	0.515		
LLM5	0.701		
Separation from Instructors (SI)		3.514	14.644
SI6	0.701		
SI7	0.768		
SI8	0.599		
SI9	0.661		
Separation from Peers (SP)		3.291	13.712
SP10	0.632		
SP11	0.761		
SP12	0.656		
Technological Challenges (TC)		3.163	13.179
TC13	0.510		
TC14	0.591		
TC15	0.711		
TC16	0.727		
TC17	0.687		
TC18	0.742		
Lack of Two-way Communication (LTwC)		3.053	12.723
LTwC19	0.579		
LTwC20	0.609		
LTwC21	0.802		
LTwC22	0.819		
LTwC23	0.810		
LTwC24	0.738		

### 4.3 Multiple linear regression analysis

The quantitative analyses demonstrated significant associations between EFL students’ online learning anxiety and various factors, including lack of learning motivation, separation from instructors, separation from peers, technological challenges, and lack of two-way communication. The analysis met all statistical assumptions, including the normal distribution of residuals, and the means, standard deviations, and correlation coefficients are presented in Table 7.

**Table 7:** Descriptive statistics and correlation coefficients of variables (*n* = 899).

	Variables	Descriptives		Correlation Coefficients					
		*M*	*SD*	OLA	LLM	SI	SP	TC	LTwc
**Dependent Variable**	OLA^a^	14.202	5.234	–					
**Independent variables**	LLM^b^	14.702	4.752	.424**	–				
	SI^b^	10.610	3.665	.380**	.645**	–			
	SP^b^	7.978	2.992	.434**	.543**	.676**	–		
	TC^b^	17.734	5.251	.435**	.614**	.648**	.698**	–	
	LTwC^b^	17.902	6.023	.383**	.600**	.678**	.681**	.770**	–

**p < .01.

Note:

a OLA = Online Learning Anxiety.

b LLM = Lack of Learning Motivation; SI = Separation from Instructors; SP = Separation from Peers; TC = Technological Challenges; LTwC = Lack of Two-way Communication.

Multiple linear regression was conducted to determine the best linear combination of lack of learning motivation, separation from instructors, separation from peers, technological challenges, and lack of two-way communication for predicting online learning anxiety among Chinese EFL students enrolled in the college English course. After using the “enter” regression method, the results revealed that the combination of lack of learning motivation, separation from peers, and technological challenges significantly predicted online learning anxiety among Chinese EFL students in the college English course, *F* (5, 893) = 59.624, *p* < .05, with each variable significantly and positively contributing to the prediction (*p* < .05). However, while separation from instructors also positively contributed to the prediction, it was not significant (β = 0.004, *p* > .05) (see Table 8). The beta weights presented in Table 8 indicated that lack of learning motivation, separation from peers, and technological challenges contributed the most to predicting online learning anxiety among Chinese EFL students in the college English course. It means that greater levels of lack of learning motivation, separation from peers, and technological challenges were associated with higher levels of online learning anxiety. In addition, lack of two-way communication showed a negative association with online learning anxiety, but it was not statistically significant (β = −0.022, *p* > .05).

**Table 8:** Multiple linear regression: important statistics (*n* = 899).

	Variables	*R*	*R*^*2*^	Adjusted *R*^*2*^	*F* (5, 893)	Beta	*t*	*P value*	Tolerance	VIF	Durbin-Watson
**Dependent Variable**	**OLA**^a^	0.500	0.250	0.246	59.624***						
**Independent variables**	**(Constant)**					5.046	8.805***	0.000			
	**LLM**^b^					0.239	5.356***	0.000	0.510	1.960	
	**SI**^b^					0.004	0.066	0.947	0.397	2.518	
	**SP**^b^					0.370	4.679***	0.000	0.410	2.437	
	**TC**^b^					0.171	3.396**	0.001	0.329	3.036	
	**LTwC**^b^					−0.022	−0.502	0.616	0.334	2.996	
											1.927

*p < .05, **p < .01, ***p < .001.

Note:

a OLA = Online Learning Anxiety.

b LLM = Lack of Learning Motivation; SI = Separation from Instructors; SP = Separation from Peers; TC = Technological Challenges; LTwC = Lack of Two-way Communication.

Furthermore, the Durbin-Watson test was conducted to assess the autocorrelation among error terms, and it revealed that the error terms had little autocorrelation, as indicated by a Durbin-Watson value of 1.927, which is close to 2. The R-square value of the multiple linear regression model was 0.250, indicating that the model explained 25% of the variance in online learning anxiety among Chinese EFL students.

Table 8 displays the beta coefficients for the five predictor variables: lack of learning motivation, separation from instructors, separation from peers, technological challenges, and lack of two-way communication. The beta coefficients reflect the slope values of each predictor variable, which are 0.239, 0.004, 0.370, 0.171, and −0.022, respectively. These coefficients indicate the extent of each predictor variable's effect on Chinese EFL students' online learning anxiety. The multiple linear regression equation for predicting Chinese EFL students' online learning anxiety in the college English course is Y = 5.046 + 0.239 × *X*_*1*_ + 0.004 × *X*_*2*_ + 0.370 × *X*_*3*_ + 0.171 × *X*_*4*_ − 0.022 × *X*_*5*_ + *ϵ,* where *X*_*1*_, *X*_*2*_, *X*_*3*_, *X*_*4*_, and *X*_*5*_ represent the predictor variables of lack of learning motivation, separation from instructors, separation from peers, technological challenges, and lack of two-way communication, respectively. In addition, *ϵ* represents the residual term, accounting for unexplained variability in the model.

## 5 Discussion

### 5.1 Anxiety levels of Chinese EFL students in the online college English course

The shift to online learning, especially in the post-COVID-19 era, has brought about significant changes in the learning environment, leading to increased anxiety levels. The present study examined the extent of online learning anxiety among Chinese EFL students enrolled in the college English course. The results of the descriptive analysis indicate that online learning anxiety is a prominent issue among Chinese EFL students. These robust descriptive findings align with prior empirical research conducted in China and other countries, which consistently demonstrate the presence of anxiety symptoms among college students in online learning environments [55,56]. Thus, it is evident that online learning anxiety is a pervasive concern for college students worldwide. The prevalence of online learning anxiety among Chinese EFL students underscores the importance of addressing this issue within educational settings. As Chinese EFL students widely experience online learning anxiety, a psychological survey or one-on-one counseling could be conducted reasonably based on students’ grades and gender. More efforts should be dedicated to enhancing the well-being of students vulnerable to mental health risks [6]. The interviews conducted in this study provided evidence for the levels of anxiety reported.

If I were to rate my anxiety on a scale of ten, it would be a solid nine. It’s challenging for me to study online because the environment is not conducive to learning. Learning online itself is already stressful enough, but the anxiety caused by my parents is even more significant than the difficulty of studying. Sometimes, when stressed about my coursework, I want my parents to understand my situation rather than blaming or pushing me. It would mean a lot if they could be more understanding and supportive. I want to feel like they get where I’m coming from. I really wish to be understood (Interviewee-B).

### 5.2 Factors contributing to anxiety among EFL students in the online college English course

This study examined the factors influencing online learning anxiety among EFL students in the college English course during the post-COVID-19 era. Our exploratory factor analysis results revealed five factors contributing to online learning anxiety: lack of learning motivation, separation from instructors, separation from peers, technological challenges, and lack of two-way communication. Overall, our findings are generally consistent with the existing body of research [7,56] and contribute to expanding Keegan's theoretical framework on online education. These results strengthen the validity of our findings and provide additional evidence for the influence of these factors on online learning anxiety among EFL students.

### 5.3 The relationship between the factors and online learning anxiety in the online college English course

The study explored the relationships between the factors and online learning anxiety through multiple linear regression. The findings indicate that students’ lack of learning motivation is a significant factor contributing to online learning anxiety. It is consistent with previous research that highlights the interaction between foreign language learning motivation and anxiety, ultimately influencing English language learning performance. Therefore, it is evident that a lack of learning motivation is crucial in affecting learning outcomes and leading to anxiety. These results align with most previous findings, indicating that a lack of motivation can cause anxious behaviors.

Additionally, the physical separation between instructors and students in an online learning environment contributes to increased anxiety among learners. The heightened anxiety is attributed to students’ difficulty asking questions or seeking timely assistance. This finding corresponds with previous studies that have demonstrated high levels of learning anxiety among students in such contexts [32,57,58].

Furthermore, the separation from peers is another significant factor contributing to higher anxiety levels among EFL students in an online learning environment. It can be attributed to the importance of learner-learner interactions and peer support in shaping individual academic emotions. In a traditional classroom, students can seek help and encouragement from their peers when encountering various challenges, such as understanding complex English text, overcoming language barriers, or engaging in speaking activities through interactive discussions. However, in online learning environments, students frequently experience a sense of isolation from their peers, resulting in a feeling of disconnection and increased anxiety. This finding aligns with the research conducted by Hurd [59], who demonstrated that the isolated context and the physical absence of instructors and peers in distance education might intensify foreign language anxiety. The finding could also be evidenced by the interview.

Before the pandemic, I would talk to my friends and classmates face-to-face when I encountered trouble. I’m a person who enjoys sharing everything that comes up, whether it’s exciting or sad. I told my friends about it and shared my joys and sorrows. Sometimes, when I was stressed, confused, or anxious, I would go for walks or visit nearby cities with my classmates and friends to relax. Additionally, I can talk to my friends about unhappiness, and talking with good friends helps clear my mind. It keeps me from feeling lost, confused, or anxious. However, it was nearly impossible to see my friends during the pandemic (Interviewee-D).

Likewise, the study reveals that technological challenges significantly impact students' anxiety toward online learning. This result confirms previous investigations that have also identified technological challenges as a contributing factor to anxiety in online learning contexts [7,60]. Furthermore, the finding is consistent with the research conducted by J.S. Barrot et al. [14], who reported that students’ anxiety stemmed from unfamiliarity with new learning platforms and technical issues.

The findings of this study reveal an intriguing insight regarding the relationship between lack of two-way communication and online learning anxiety among EFL students. Interestingly, our results contradict some prior research conducted in the Western context, showing that lack of two-way communication causes or increases anxiety among students [61]. Keegan's theory also underscored the significance of “two-way communication,” stating that students should be able to actively engage in dialogue rather than being solely passive recipients. However, the findings indicate a lack of two-way communication between instructors and students was associated with reduced online learning anxiety among EFL students. One possible explanation for the negative correlation in this study is that Chinese cultural values are shaped by three major belief systems: Confucianism, Buddhism, and Taoism. These values may influence how Chinese people perceive themselves and their relationships with others, influencing how they behave and respond to the world [62]. It could also be verified by the previous research, which indicated that East Asian learners' unwillingness to communicate in classes is frequently attributed to the influence of the Confucian Heritage Culture [63]. In Chinese culture, students are traditionally accustomed to assuming a more passive role as lecture listeners, which may explain why the lack of two-way communication does not elicit anxiety among Chinese students. The insights derived from the interview data further support this evidence.

I’m the type of student who prefers to take a more passive role in class. I feel more at ease simply listening to the teacher. But I must admit that I get a little nervous when I think I might be called to answer questions. That can cause anxiety. So, the absence of two-way communication during instruction does not cause me anxiety (Interviewee-I).

As discussed, the multiple regression results show that lack of learning motivation, separation from peers, and technological challenges are the most influential factors in causing online learning anxiety among Chinese EFL students in the college English course. Therefore, several measures can be taken to reduce anxiety and promote successful online learning. Firstly, to address the issue of anxiety stemming from students' low motivation in online learning, it is recommended that Classroom Action Research be used to enhance both the quality of English teachers' performance and students' motivation and achievement in English language acquisition. In addition, incorporating online platforms can boost EFL students’ motivation to improve their English proficiency [64]. Secondly, students are expected to interact with peers or a language partner, for instance, by connecting and interacting with peers through discussion forums and online blogs [65], which will reduce the feeling of isolation. Thirdly, regarding technical issues, students can use technological aptitude enhancement and help-seeking as feasible strategies [14].

## 6 Research limitations

Limitations of this study and areas for future improvement in examining the factors affecting online learning anxiety in the college English course were also identified. Firstly, the findings are context-dependent and do not generalize to the global population. Secondly, as the factors causing anxiety are multifaceted, the factors explored in this study do not fully explain the online learning anxiety in the college English course, and there are some other factors, such as online learning experiences, learning style, and course design, that should be taken into consideration. Hence, further research should be conducted to explore additional variables that influence students’ online learning anxiety and to address these factors to reduce anxiety and enhance the quality of online learning experiences.

## 7 Conclusion

The current study investigated the overall state of Chinese EFL students' online learning anxiety in the college English course and presented an empirical examination of the factors and EFL students' online learning anxiety guided by Keegan's online education theory. It was found that more than half of the EFL students reported different degrees of anxiety, ranging from mild to moderate or severe levels. This study pinpointed the factors that contribute to online learning anxiety among college EFL students. These findings emphasize the significance of identifying these factors in predicting students' anxiety. Additionally, the study expanded upon Keegan's theoretical framework and added value to the existing literature on online education by identifying the factors associated with online learning, specifically in the Chinese context during the post-COVID-19 pandemic era. The implications of this study are twofold. On the one hand, it enhances our comprehension of the English learning anxiety experienced by students. On the other hand, it holds theoretical and pedagogical importance in addressing the factors contributing to online learning anxiety in the college English course. Finally, this study proposes several suggestions for alleviating online learning anxiety among EFL students and provides effective interventions to support students' mental health and well-being.

## Ethics statement

Approval was obtained from the School of Foreign Languages ethics committee, Huazhong University of Science and Technology (the ethical approval of Research Project Protocol code: #2022-SFL/IRB-0820).

## Funding information

The work was supported by Hubei Provincial Teaching and Research Project under grant (Number: 2022452).

## Data availability statement

Data will be made available on request.

## Additional information

No additional information is available for this paper.

## CRediT authorship contribution statement

**Renzhong Peng:** Writing – review & editing, Writing – original draft, Methodology, Funding acquisition, Formal analysis, Conceptualization. **Shiying Wang:** Writing – original draft, Software, Investigation, Formal analysis, Data curation. **Na Liu:** Writing – review & editing, Supervision, Resources, Project administration.

## Declaration of competing interest

The authors declare that they have no known competing financial interests or personal relationships that could have appeared to influence the work reported in this paper.
